# Candidate Genes Detected in Transcriptome Studies Are Strongly Dependent on Genetic Background

**DOI:** 10.1371/journal.pone.0015644

**Published:** 2011-01-25

**Authors:** Pernille Sarup, Jesper G. Sørensen, Torsten N. Kristensen, Ary A. Hoffmann, Volker Loeschcke, Ken N. Paige, Peter Sørensen

**Affiliations:** 1 Department of Biological Sciences, Aarhus University, Aarhus, Denmark; 2 Department of Terrestrial Ecology, National Environmental Research Institute, Aarhus University, Silkeborg, Denmark; 3 Department of Genetics and Biotechnology, Danish Institute of Agricultural Sciences, Aarhus University, Tjele, Denmark; 4 Department of Genetics and Centre for Environmental Stress and Adaptation Research, The University of Melbourne, Melbourne, Australia; 5 Department of Animal Biology, School of Integrative Biology, University of Illinois, Urbana, Illinois, United States of America; Virginia Tech Virginia, United States of America

## Abstract

Whole genome transcriptomic studies can point to potential candidate genes for organismal traits. However, the importance of potential candidates is rarely followed up through functional studies and/or by comparing results across independent studies. We have analysed the overlap of candidate genes identified from studies of gene expression in *Drosophila melanogaster* using similar technical platforms. We found little overlap across studies between putative candidate genes for the same traits in the same sex. Instead there was a high degree of overlap between different traits and sexes within the same genetic backgrounds. Putative candidates found using transcriptomics therefore appear very sensitive to genetic background and this can mask or override effects of treatments. The functional importance of putative candidate genes emerging from transcriptome studies needs to be validated through additional experiments and in future studies we suggest a focus on the genes, networks and pathways affecting traits in a consistent manner across backgrounds.

## Introduction

In *Drosophila* an increasing number of whole genome expression studies relating gene expression to genetic differences in stress resistance traits and longevity have now been carried out [1], [2]–[7]. These studies are focused on identifying candidate genes and genetic networks of importance for lifespan and resistance to stressful conditions including heat, cold and desiccation resistance. However, with recent advances in transcriptomics the number of putative candidate genes is accumulating much faster than what can be verified in much detail. Few candidate genes detected in *Drosophila* studies have so far been validated by studies on knock-out or over-expression lines or by functional genomics studies using sequencing or an association mapping SNP approach (for exceptions see) [7]. Although whole genome expression studies have proved fruitful in some organisms [8]–[11], it is still unclear to what degree candidate genes identified in transcriptomic studies will be valuable and relevant for candidate gene identification [12].

As multiple whole genome transcriptomic studies aiming at identifying genes and pathways explaining variation in similar traits become available, it becomes possible to evaluate the repeatability of changes in transcriptomic patterns across studies. Any similarity among studies might well depend on the effect of 1) genetic background and standing genetic variation - there might be more than one way to obtain similar phenotypes, 2) inbreeding/genetic drift effects on genome wide gene expression patterns, and 3) impacts of environmental conditions that may vary between laboratories.

Two strategies are mainly used to detect candidate genes in *D. melanogaster*. Lines can be selected in the laboratory for increased stress resistance/longevity and compared to control flies that differ in the phenotype of interest. Alternatively, phenotypic variation in traits of interest in highly inbred isogenic lines can be associated to gene expression in these lines. Results from the different studies make it possible to investigate to what degree genetic background or inbreeding influence the lists of candidate genes detected.

In this paper we compare the gene lists from 4 different whole genome transcriptome studies on *D. melanogaster* investigating overlapping traits [1], [4]–[6]. In order to further evaluate whether inbreeding *per se* influences patterns, we included two studies on the effect of inbreeding on the transcriptome [13], [14]. We found a much larger proportion of significant overlap between traits within genetic background than within similar traits investigated in different genetic backgrounds. There was also a tendency for inbreeding to affect transcription in a directional manner. In the light of our results we conclude that transcriptome studies should be interpreted cautiously and that it is advisable where possible to validate the functional relationship between candidate genes from transcriptome studies and the specific trait in question. This also has implications for the emerging transcriptome studies in non-model species [15], [16], where functional validation of candidate genes will be difficult. Additionally, studies could be designed to include a focus on networks of genes being differentially expressed across several independent genetic backgrounds.

## Materials and Methods

We reanalysed and compared gene expression datasets from six studies on gene expression in *D. melanogaster* [1], [4]–[6], [13], [14]. Table 1 summarises the traits and sexes investigated in these studies. In all studies global gene expression was assayed using Affymetrix Drosophila (version 1 or 2) microarrays. Data from Ayroles *et al.* [4] was reanalysed with sexes separate (data kindly provided by T.F.C. Mackay). The array data was analysed using R (version 2.9.0) (http://www.r-project.org/) based applications. The raw data was GC-RMA normalised with the BIOCONDUCTOR application for R [17] as implemented in the ‘Affy’ package for R (version 1.22.1). With respect to the data from the study of Ayroles *et al.* [4], the *t*-test statistics were generated based on the association between the organismal phenotypes and the expression data from information on 40 inbred lines. We used the gene list generated in [14] while the remaining data sets were analysed contrasting the selected or inbred lines with control lines.

**Table 1:** Summary of the transcriptomic studies included in the analyses.

Authors	Isogenic/inbred or outbred lines	Sex	Genetic background	Longevity	Chill comarecovery	Locomotor activity	Mating speed	Starvation resistance	Fitness	Inbreeding	Heat 30°C	Cold resistance	Desiccation resistance	Heat resistance	Heat knock down
Telonis-Scott *et al.* [5]	O	F	a		x										
Sørensen *et al.* [6]	O	F	b	x				x			x	x	x	x	x
Ayroles *et al.* [4]	I	F/M	c	x	x	x	x	x	x						
Sarup *et al.* [1]	O	M	b	x											
Ayroles *et al.* [14]	I	M	d							x					
Kristensen *et al.* [13]	I	M	b							x					

Sexes are indicated by F: female and M: male, I: studies using isogenic/inbred lines and O: studies using outbred lines, studies sharing genetic background are denoted by similar letters and traits investigated are marked for each study. For further details see the original papers.

Significance of all datasets was re-evaluated following [4] with a cut off at P<0.01 and no FDR correction to equalise the methodology. The resulting lists of significant genes were used as the basis for analyses. To compare among different versions of Affymetrix gene chips, Entrez IDs were used as the common identifier for all genes. We identified the overlap among gene lists and estimated the probability that the overlap of differentially expressed genes varied from the number expected by chance using Monte Carlo simulations. The empirical P-value for the observed overlap of genes among the different treatments was determined using simulations. In each simulation, the gene list for each treatment was permutated and the random overlap among gene lists was recorded. This procedure was repeated 100,000 times. The empirical P-value was determined as the fraction of all permutations where the observed overlap was larger or equal to the random overlap among the gene lists.

## Results

The 253 contrasts investigated showed large differences in gene overlaps (Table 2). The generated lists of significant genes from each study contained between 165 and 1944 genes (average 528), and the overlaps ranged between 1 and 249 (average 34.8).

**Table 2:** Overlap gene lists.

	F C30 (6,b)	F CCR (5,a)	F CCR (4,c)	F Co (6,b)	F DS (6,b)	F Fit (4,c)	F H (6,b)	F KD (6,b)	F Loc (4,c)	F Long (6,b)	F Long (4,c)	F Mate (4,c)	F Starv (6,b)	F Starv (4,c)	M CCR (4,c)	M Fit (4,c)	M I (13,b)	M Loc (4,c)	M Long (4,c)	M Long (1,b)	M Mate (3,c)	M Starv (4,c)	M I (14,d)
F C30 (6,b)	202	3	17	43	42	15	50	76	10	61	4	3	64	9	16	12	10	18	8	7	19	7	5
F CCR (5,a)	NS	244	*22*	4	5	11	11	9	18	6	12	8	10	11	*16*	23	5	21	15	12	28	18	18
F CCR (4,c)	NS	*NS*	1291	20	21	71	114	43	44	27	23	51	25	78	*252*	166	23	58	39	29	233	93	43
F Co (6,b)	**<0.001**	NS	NS	187	49	20	93	71	14	48	5	2	61	2	17	12	7	17	5	5	17	9	9
F DS (6,b)	**<0.001**	NS	NS	**<0.001**	230	16	37	74	2	58	6	4	101	6	19	14	8	22	9	11	32	7	7
F Fit (4,c)	<0.05	NS	**NS**	<0.001	<0.05	602	77	41	29	16	14	19	23	52	67	*169*	18	35	13	11	66	56	32
F H (6,b)	**<0.001**	NS	<0.001	**<0.001**	**<0.001**	<0.001	834	161	25	52	24	15	61	23	134	83	17	50	30	19	75	68	48
F KD (6,b)	**<0.001**	NS	NS	**<0.001**	**<0.001**	<0.001	**<0.001**	360	11	78	8	7	102	14	40	33	9	19	23	12	44	15	14
F Loc (4,c)	NS	<0.001	**NS**	<0.001	NS	**<0.05**	NS	NS	416	1	17	4	8	15	27	35	17	*96*	26	14	69	20	29
F Long (6,b)	**<0.001**	NS	NS	**<0.001**	**<0.001**	<0.05	**<0.001**	**<0.001**	NS	212	*5*	6	101	6	20	15	9	20	*5*	*5*	23	9	6
F Long (4,c)	NS	<0.001	**NS**	NS	NS	**NS**	<0.05	NS	**<0.001**	*NS*	237	10	7	23	17	18	6	19	*50*	*7*	38	10	20
F Mate (4,c)	NS	<0.05	**<0.001**	NS	NS	**<0.001**	NS	NS	**NS**	<0.05	**<0.001**	167	3	7	33	26	4	5	13	7	*90*	16	15
F Starv (6,b)	**<0.001**	<0.05	NS	**<0.001**	**<0.001**	<0.01	**<0.001**	**<0.001**	NS	**<0.001**	NS	NS	278	*9*	30	20	9	25	11	8	27	11	8
F Starv (4,c)	NS	NS	**<0.001**	NS	NS	**<0.001**	NS	NS	**NS**	NS	**<0.001**	**NS**	*NS*	422	69	103	21	28	39	11	72	87	29
M CCR (4,c)	NS	*NS*	***<0.001***	NS	NS	**<0.001**	<0.001	<0.001	**NS**	NS	**NS**	**<0.001**	<0.01	**<0.001**	867	196	21	88	48	33	106	136	53
M Fit (4,c)	NS	<0.05	**<0.001**	NS	NS	***<0.001***	<0.001	<0.05	**NS**	NS	**NS**	**<0.001**	NS	**<0.001**	**<0.001**	876	21	43	42	27	159	160	46
M I (13,b)	**<0.001**	NS	NS	**<0.05**	**<0.05**	<0.01	**NS**	**NS**	<0.001	**<0.001**	NS	NS	**<0.05**	<0.001	<0.05	<0.05	216	9	11	14	15	21	*20*
M Loc (4,c)	<0.05	<0.05	**NS**	<0.05	<0.001	**NS**	<0.05	NS	***<0.001***	<0.01	**<0.01**	**NS**	<0.001	**<0.05**	**<0.001**	**NS**	NS	611	22	19	49	24	39
M Long (4,c)	NS	<0.05	**NS**	NS	NS	**NS**	NS	<0.001	**<0.001**	*NS*	***<0.001***	**<0.01**	NS	**<0.001**	**<0.001**	**<0.01**	NS	**NS**	439	*23*	63	58	35
M Long (1,b)	**NS**	<0.05	NS	**NS**	**<0.05**	NS	**NS**	**NS**	NS	**NS**	*NS*	NS	**NS**	NS	<0.05	NS	**<0.01**	NS	*<0.001*	350	46	17	16
M Mate (3,c)	NS	NS	**<0.05**	NS	NS	**NS**	NS	NS	**NS**	NS	**NS**	***<0.001***	NS	**NS**	**NS**	**<0.01**	NS	**NS**	**NS**	NS	1965	58	62
M Starv (4,c)	NS	<0.05	**<0.001**	NS	NS	**<0.001**	<0.001	NS	**NS**	NS	**NS**	**<0.001**	*NS*	***<0.001***	**<0.001**	**<0.001**	<0.001	**NS**	**<0.001**	NS	**NS**	594	51
M I (14,d)	NS	<0.05	NS	NS	NS	<0.05	<0.001	NS	<0.001	NS	<0.001	<0.001	NS	<0.001	<0.001	<0.05	*<0.001*	<0.001	<0.001	NS	NS	<0.001	500

Numbers above the diagonal denote overlapping genes between lists, below the diagonal are P-values, and on the diagonal is the number of unique genes in the lists. NS, non-significant. The letters in parentheses specify genetic background (see Table 1). Comparisons between same traits are in italics and same genetic background are in bold. M: Males, F: Females, C30: Heat 30°C, CCR: Chill coma recovery, Co: Cold resistance, DS: Desiccation resistance, Fit: Fitness, H: Heat resistance, KD: Heat knock down, Loc: Locomotor activity, Long: Longevity, Mate: Mating activity, Starv: Starvation resistance, I: Inbreeding. Numbers denote paper codes (see Table 1). Letters denote genetic background (see Table 1).

Of the 253 individual contrasts, 113 were significantly larger than expected by chance. One noticeable result was the lack of significant overlap among studies looking for candidate genes for the same traits (Table 2). This was true for starvation resistance, chill coma recovery time and female lifespan. Only for male longevity did we detect a significant gene overlap between the studies of Sarup *et al.* [1] and Ayroles *et al.* [4]. In general the overlap was not larger among similar traits (chill coma recovery, starvation and longevity/life span) than among traits not expected to be functionally correlated.

A clear pattern was the apparent similarity among sexes in cases where both sexes were investigated for the same trait in the same genetic background (5 significant overlaps out of 7 comparisons); the only exception was longevity where we did not find a significant overlap within the study of Ayroles *et al.* [4] or between the genes that were found studying males [1] and females [6].

### Genetic background

**Figure 1:**
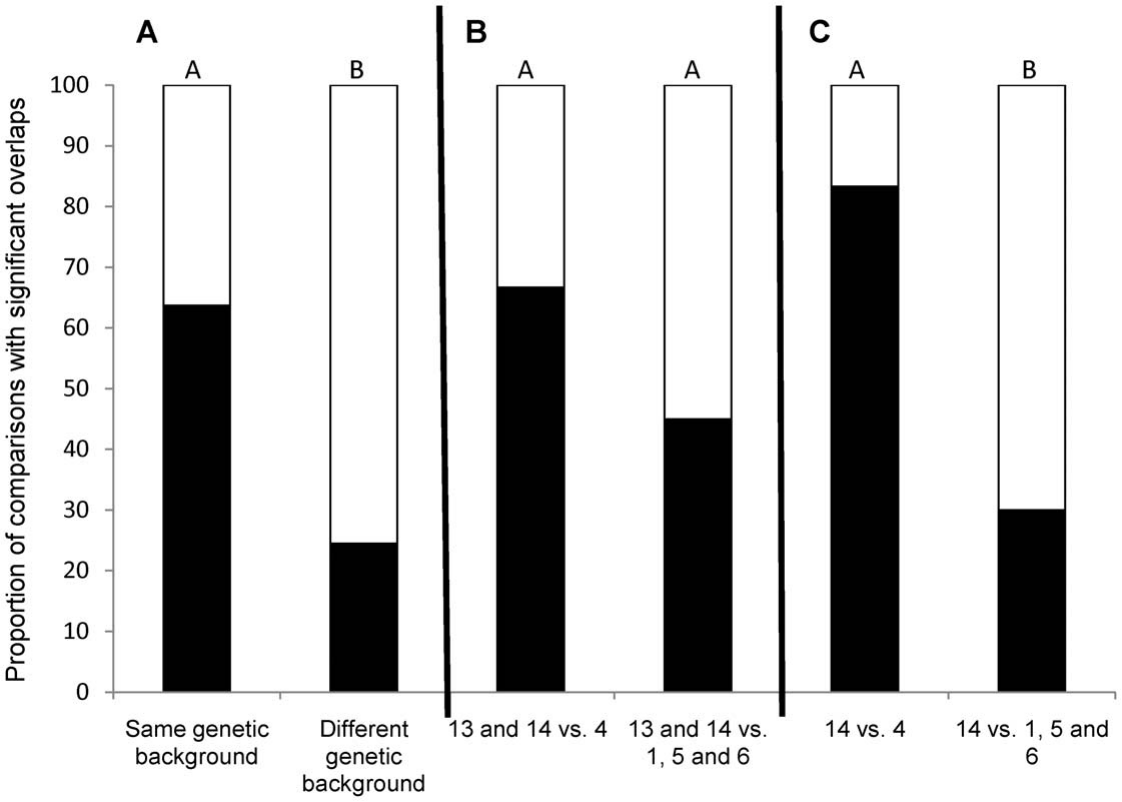
Genetic background and inbreeding effects on the number of significant overlaps among gene lists. The figure depicts the proportion of gene list comparisons that results in significant overlaps (black: significant, white: non-significant). Different letters denote proportions that are significantly different. **A**: 102 comparisons between gene lists from studies with the same genetic background and 151 comparisons between gene lists from studies with different genetic backgrounds. **B**: 24 gene list comparisons: Kristensen *et al.* [13] and Ayroles *et al.* [14] *vs.* Ayroles *et al.* [4] and 20 gene list comparisons: Kristensen *et al.* [13] and Ayroles *et al.* [14] *vs.* Sørensen *et al.* [6], Telonis-Scott *et al.* [5] (1) and Sarup *et al.* [1]. **C**: 12 gene list comparisons: Ayroles *et al.* [14] *vs.* Ayroles *et al.* [4] and 10 gene list comparisons: Ayroles *et al.* [14] (5) *vs.* Sørensen *et al.* [6] , Telonis-Scott *et al.* [5] (1) and Sarup *et al.* [1].

We found a high number of overlaps of candidate gene lists within the same genetic background (65 significant overlaps out of 102 comparisons) compared to the overlaps between genetic backgrounds (37 out of 151). This difference in the frequency of overlaps was larger than expected by chance (Figure 1**A**, χ^2^ = 19.3, P<0.001).

### Inbreeding

The proportion of significant overlaps between the study that associates organismal traits with gene expression in inbred lines [4] and the studies of inbreeding effects on the transcriptome [13], [14] (16 out of 24) was higher than the proportion of significant overlaps between the studies of inbreeding effects on the transcriptome and the studies on outbred lines [1], [5], [6] (9 out of 20), although this difference was not significant (Figure 1**B**). However, the studies of Kristensen *et al.* [13], Sørensen *et al.* [6] and Sarup *et al.* [1] share a common genetic background, so this comparison was confounded by effects of genetic background and inbreeding. Omitting the study of Kristensen *et al.* [13], there were 12 comparisons that associate organismal traits with gene expression in inbred lines [4] and Ayroles *et al.* [14] with 10 significant overlaps, and 10 comparisons between the remaining studies [1], [5], [6] and Ayroles *et al.* [14] with 3 significant comparisons. There was a significant difference between the study using inbred lines [4] and those using outbred lines [1], [5], [6] in the proportion of significant overlaps with the study on the effects of inbreeding depression on the trascriptome [14] (Figure 1**C**, χ^2^ = 6.7, P<0.01).

## Discussion

### Genetic background

If genetic background has a large impact on the list of candidate genes generated from full genome transcriptomic studies, we expect a high degree of overlap between traits in common genetic backgrounds. This is actually what we observe, as contrasts performed on the same genetic background (Table 1) [1], [4], [6], [13] have a high proportion of significant overlaps (Table 2) independent of whether the same traits or different traits are considered. Genetic background effects are a likely cause of this discrepancy although other factors such as laboratory-specific environmental conditions and inbreeding/genetic drift might also contribute. This points to caution in extrapolating results from one transcriptomic study to another and also highlights the general importance of genetic background in evolutionary studies (see also) [18], [19]. Based on our findings we suggest that future studies aiming to identify candidate genes/pathways should consider validating detected genes/pathways across different backgrounds.

The population-specific nature of candidate genes detected via transcription studies might reflect the fact that a candidate gene can only be detected in association or selection studies if there is variation in relevant loci either in the base population or arising from mutations during the selection/line establishment process. Moreover due to genetic drift, allelic variation present within the base population might differ between replicate lines in selection experiments or between inbred lines often used in *Drosophila* association studies. Thus ‘false candidate genes’ may be detected due to genetic drift. To rule out this explanation/hypothesis, effective population sizes should be high in base populations/replicate lines.

### Inbreeding

A high level of inbreeding results in increased homozygosity and expression of deleterious recessive alleles not expressed to the same extent in large natural populations. Inbreeding depression is known to affect multiple traits including lifespan and stress resistance traits in *Drosophila* [20]–[22] and inbreeding *per se* can also result in changes in gene expression of hundreds of genes [13], [14], [23], [24].

Ayroles *et al.* [4] associated organismal phenotypes (chill coma recovery, starvation, lifespan, fitness, mating time and locomotion) with gene expression in 40 highly inbred *D. melanogaster* lines. Based on these associations, a number of candidate genes for the investigated traits were proposed. A future challenge is to determine whether some alleles of importance for the traits in question have been purged or lost due to drift during the inbreeding process, and whether variation in organismal phenotype and transcription patterns might be partly due to some lines suffering more from inbreeding depression than others.

We need more studies to improve our understanding of the underlying genetic structure of stress resistance and longevity traits and to be able to determine to what extent the overlap among gene lists from studies of the same trait in the same sex is affected by different genetic backgrounds, the influence of inbreeding/genetic drift on the transcriptome or a combination of these factors. More studies are required which investigate the response of the transcriptome to selection in both sexes as such studies could help elucidating whether the large overlap between sexes in Ayroles *et al.* [4] (Table 2) was caused by genetic background and/or inbreeding. Finally, we need to test whether the few genes that show consistent changes across studies are those most likely involved in trait variation. This could be achieved by functional studies of those genes compared to genes specific to particular studies and genetic backgrounds.
